# Fixation of a Proximal Humeral Fracture Using a Novel Intramedullary Cage Construct following a Failed Conservative Treatment

**DOI:** 10.1155/2017/4347161

**Published:** 2017-02-01

**Authors:** John Macy

**Affiliations:** Copley Hospital, Morrisville, VT 05661, USA

## Abstract

A majority of proximal humeral fractures are preferably treated conservatively. However, surgical management may be beneficial in proximal humeral fractures with significant displacement or angulation. Unfortunately, the complication rates associated with current surgical procedures for fracture fixation, ORIF and IM devices, can be unacceptably high. A new technology, termed the PH Cage, addresses the technical limitations associated with current technologies available for fixation of proximal humeral fractures. It allows for intramedullary fixation of a PH fracture and provides direct load bearing support to the articular surface and buttresses the medial column during healing. We are presenting our first experience with the PH Cage for the fixation of a PH fracture, which had previously failed conservative management.

## 1. Introduction

Optimal management of proximal humeral (PH) fractures continues to be controversial. Since 71% of proximal humerus fractures occur in patients over sixty years in age, conservative management is currently preferred in a majority of these patients [1]. However, clinical studies have shown that nonoperative treatment of certain fracture types can significantly lower functional outcomes in some patients [2]. In spite of this evidence, surgical intervention may not be recommended because of potentially high complication rates associated with existing technologies for PH fracture fixation. However, as the degree of displacement and instability increases, conservative management of fractures results in suboptimal outcomes [3]. Surgical intervention may be preferred to optimally manage these significantly displaced two-, three- and four-part fractures.

Surgical techniques are constantly evolving to manage PH fractures either through reconstruction (with pins, plates, screws, and IM nails) or prosthetic replacement options (hemiarthroplasty and reverse shoulder arthroplasty). Of the current surgical treatments available, the evolution of locking plate technologies has increased the incidence of surgical interventions to fix PH fractures. Proximal humeral locking plates are indicated for the fixation of certain displaced two-, three-, and four-part PH fractures. Locking plates provide biomechanical strength and stability for restoring and fixing a fracture, especially for valgus impacted fractures. However, the overall clinical benefit of locking plates for PH fracture fixation is controversial, both in their ability to treat complex PH fractures and in the predictability of patient outcomes. When compared to conservative treatment in elderly patients, a recent randomized clinical trial showed better radiographic outcome with open reduction and internal fixation (ORIF) but statistically equivalent functional outcomes for patients with three-part fractures [4]. Additionally, complication rates associated with locking plate technologies can be unacceptably high.

Complication rates as high as 50% have been reported in literature for locking plates with associated revision rates at approximately 15% [5]. Complications include intra-articular screw penetration, hardware failure, subacromial impingement, varus collapse, and osteonecrosis [6]. To ensure optimal clinical outcomes, the current consensus is that the restoration of the medial calcar, metaphyseal buttressing, and anatomic reduction of the tuberosities are key [7]. The use of intramedullary fibular strut allografts has been reported to aid in reduction, as well as to provide buttress support to the medial column for patients with osteoporotic bone with good clinical outcomes [8]. However, there is a need for new technology that addresses the limitations of locking plates and IM devices for the fixation of proximal humeral fractures.

The PH Cage (Conventus Orthopaedics, Maple Grove MN, USA) is an intramedullary implant available for fixation of proximal humeral fractures. The PH Cage is made from nitinol and it expands once deployed below the articular surface, thus providing medial column buttress and head support on implantation. The PH Cage is indicated for the fixation of two-, three-, and four-part fractures similar to the locking plates. It can be surgically inserted in a retrograde or antegrade/intramedullary direction, percutaneously or through a traditional open approach. The intramedullary design of this implant enables medial column support, which is often required for PH fractures. Additionally, the PH Cage design also allows for unconstrained screw fixation of the tuberosities wherever it is needed (unlike current locking plates or IM devices that are directionally constrained). This report presents our first experience and outcome following the use of the PH Cage for treatment of a proximal humerus fracture malunion with severe varus angulation.

## 2. Case Report

**Figure 1:**
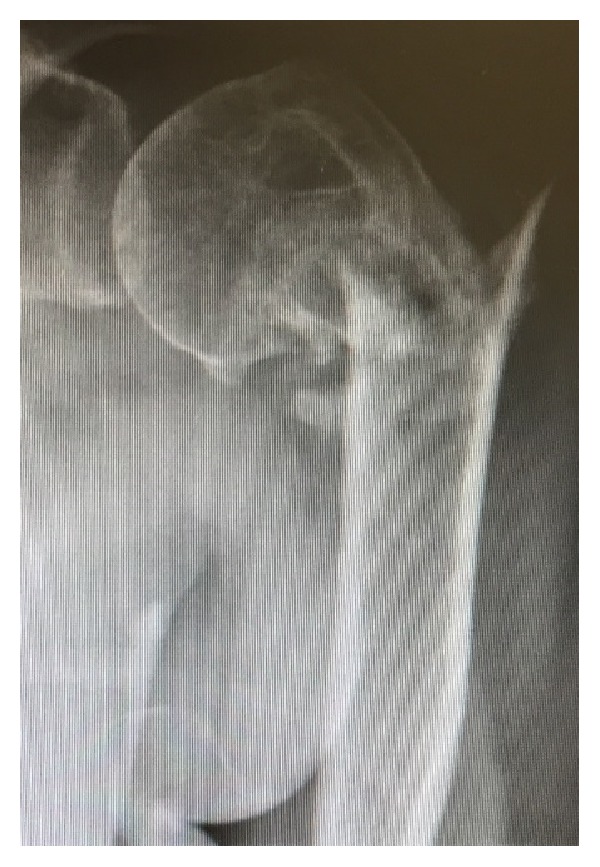
Preoperative radiograph depicting a two-part fracture of the proximal humerus. Note the varus malalignment of the head and lateral bone spike.

A 70-year-old, right hand dominant, otherwise healthy and active, female presented to our clinic three (3) months after falling onto her left shoulder. She was initially treated elsewhere nonoperatively with sling immobilization and limited physical therapy. Upon presentation to us, she complained of persistent lateral shoulder pain and limited function in her left arm. Physical examination revealed her to be neurovascularly intact with no deltoid deficiency. She had limited active motion and painful passive motion associated with crepitation. Radiographs revealed a two-part, varus-angulated malunion with a large spike of bone protruding laterally, without evidence of AVN (Figure 1). The natural history, prognosis, treatment options, potential complications, and expected outcomes for both operative and nonoperative management were reviewed with the patient. Using a shared-decision making process, she elected to proceed with surgical management.

The patient was taken to the operating room for an ORIF procedure using the PH Cage. The patient was positioned in a modified beach chair setup using a shoulder specific table and articulated arm holder for the procedure. C-arm fluoroscopy was positioned “over the top” of the patient to allow for both AP and lateral views using internal/external rotation of the arm during the procedure. An extended deltopectoral incision was used to expose the fracture. The axillary nerve was identified inferiorly and laterally as it wrapped around the humerus close to the fracture site. Since the fracture was well healed, a surgical osteotomy was required for mobilization and reduction of the fracture. A reduction jig, which resembles the contours of a locking plate, was used for initial fracture fixation. The reduction jig comes attached with an optional plate, which may be used per surgeons' discretion. Kirschner wires were used in conjunction with the reduction jig to obtain and maintain provisional reduction and fixation of the fracture. An 8 mm hole was drilled over a guide wire from the distal end of the reduction jig to approximately 5 mm below the articular surface of the head. The metaphyseal area below the head was then prepared using a tool specifically designed to break down the intramedullary cancellous bone without disrupting the subcortical bone. The PH Cage was then inserted in a retrograde manner, deployed, expanded, and then locked in position. The PH Cage is available in three different sizes: small, medium, and large. For this patient, a medium PH Cage was indicated.

The distal end of the PH Cage was locked to the plate using two 28 mm screws. At the proximal end, three screws were used to secure the fracture fragments to the PH Cage and the plate construct. One of these screws was a kickstand screw across the fracture line that was stabilized by the plate and the PH Cage on either side of the fracture. One additional screw was added outside of the plate to secure the greater tuberosity to the PH Cage construct. A titanium washer was added to that screw to buttress the screw head as well as to augment fixation of the rotator cuff. Intraop fluoroscopy confirmed adequate reduction and hardware position. The entire construct moved well as a unit under direct visualization and fluoroscopic control.

**Figure 2:**
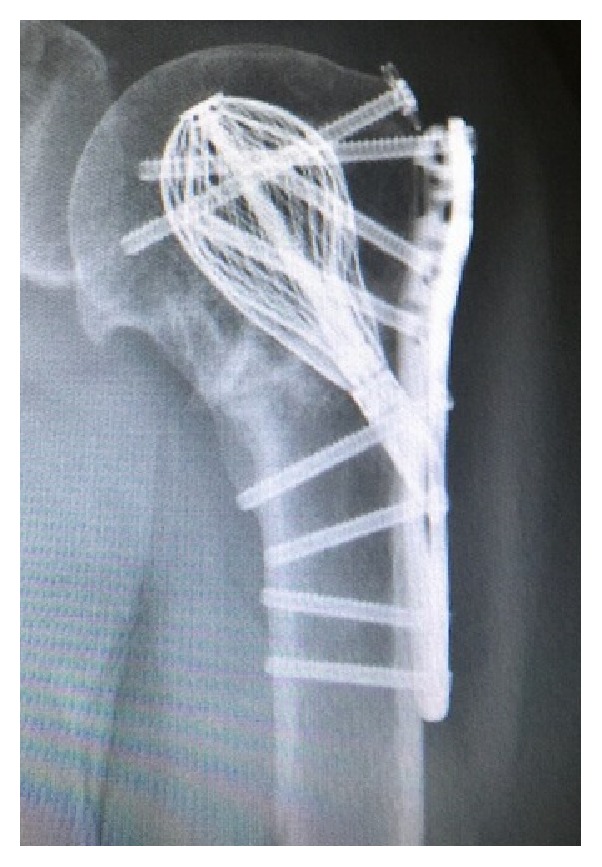
Radiograph at 6-month follow-up after fixation using a PH Cage and side plate construct.

**Figure 3:**
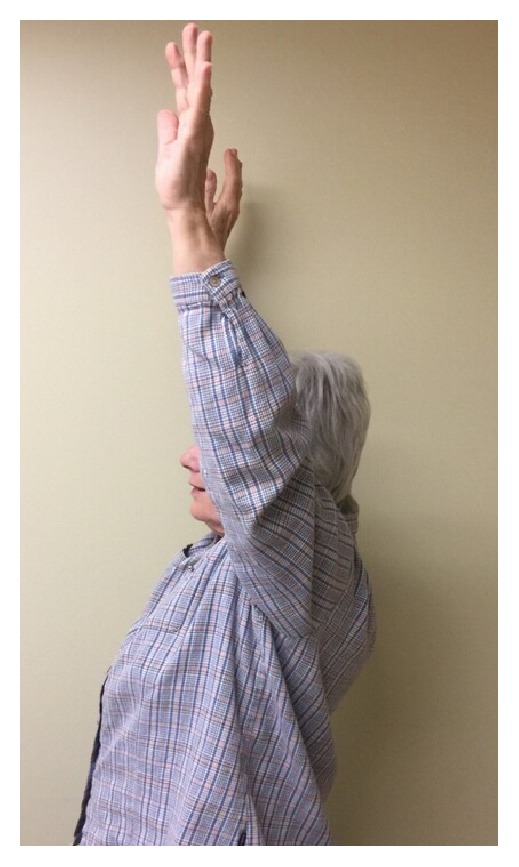
Patient exhibiting excellent range of motion at 6-month postop follow-up.

Postoperatively, the patient was immobilized in a sling for 6 weeks. She was started on pendulum/Codman exercises on POD1 and formal physical therapy involving gentle passive motion at week 1. The rehab protocol was advanced to active motion after 6 weeks and strengthening after 12 weeks. There were no intraoperative or postoperative complications. Her most recent follow-up X-rays obtained at 6 months after operation revealed a well healed fracture with anatomical alignment and no hardware complications or AVN (Figure 2). The PH Cage maintained the head and the screws in position, thus preventing varus collapse or intraarticular screw penetration. The patient exhibited excellent range of motion, strength, and function. She had no significant pain at last follow-up (Figure 3).

## 3. Discussion

Locking plate technologies are preferentially used for surgical fixation of proximal humeral fractures but the associated complication rates can be unacceptably high. Clinical studies have shown complication rates as high as 50% following PH fracture fixation using locking plates [5] and other IM devices. Many of these complications require reintervention to address either the soft tissue or implant-related issues. The primary implant-related complications reported for locking plates are screw perforation of the humeral head and varus collapse of the fracture [6]. As locking plates have evolved, some of these complications have been addressed but there are inherent limitations in supporting varus fracture patterns using plates positioned on the lateral side of the humerus. Buttressing the medial column is key and it has been shown to be effective in providing biomechanical stability to a fracture, thus decreasing clinical complications associated with varus collapse [9]. As such the use of fibular strut allografts have been used as a potential solution to buttress the medial column, preventing varus collapse of the fracture [8]. Use of allograft struts, however, requires significant surgical dissection and potential disruption to important vascular support for fracture fragments and the inherent risks of graft failure, rejection, and disease transmission.

The PH Cage is a new technology that is able to fill the metaphyseal void created by the fracture, providing direct load bearing support below the articular surface of the humeral head. It also buttresses and supports the medial column, thus increasing the biomechanical stability of the fracture post fixation, without the need for allograft struts. The cage design also allows for unconstrained positioning of screws for tuberosity fixation. In this case, we used a standard deltopectoral surgical approach to reduce and fix the fracture using the PH Cage. The technique described is consistent with recent literature supporting the use of the PH Cage for fracture fixation [10]. The three-dimensional construct provides discretion in the number of and direction of screws used to fix the fracture fragments onto the PH Cage. In this particular case, the greater tuberosity screw was used outside the plate and the fragment was directly secured to the PH Cage construct. The design of the PH Cage locks the screw in place much like a locking plate would without limitations on the number and angle of screws.

This is a retrospective case review of one difficult proximal humerus fracture malunion that went on to anatomic healing and an excellent patient-reported outcome. The PH Cage has been used in multiple proximal humeral fracture types and settings. We are currently performing a prospective study evaluating the radiographic and clinical outcome of the PH Cage technology in comparison to existing technologies for the treatment of proximal humeral fractures.
